# Rapid Tests versus ELISA for Screening of HIV Infection: Our Experience from a Voluntary Counselling and Testing Facility of a Tertiary Care Centre in North India

**DOI:** 10.1155/2014/296840

**Published:** 2014-04-07

**Authors:** Bhanu Mehra, Sonali Bhattar, Preena Bhalla, Deepti Rawat

**Affiliations:** Department of Microbiology, Maulana Azad Medical College, New Delhi 110002, India

## Abstract

Early and accurate diagnosis of human immunodeficiency virus (HIV) infection is essential for timely identification of patients needing antiretroviral therapy and for instituting HIV prevention strategies. The primary methodology for HIV testing has shifted from enzyme linked immunosorbent assay (ELISA) to rapid diagnostic tests (RDTs) in recent years, especially in resource limited settings. However, the diagnostic performance of RDTs is a matter of concern. In the present study the performance of an RDT being used as the initial test in serial testing based algorithm for HIV diagnosis was compared with ELISA. Seven hundred and eighty-seven sera, tested at the voluntary counselling and testing facility employing a serial testing algorithm (based on SD Bioline HIV-1/2 3.0 as the first test), were subsequently tested with Microlisa-HIV for anti-HIV antibodies. The first test missed 9 HIV reactive samples and also registered 5 false positives. The sensitivity, specificity, and negative and positive predictive values of the first test were 77.5%, 99.3%, and 98.8% and 86.1%, respectively, taking ELISA as the standard test. Our study highlights that RDTs fare poorly compared to ELISA as screening assays and that reactive results by RDTs need to be confirmed by western blot for a positive serodiagnosis of HIV infection.

## 1. Introduction

Approximately 35.3 million people across the world are infected with human immunodeficiency virus (HIV) [1]. Early and accurate knowledge of HIV serostatus of an individual is the cornerstone of HIV prevention and therapeutic intervention. In addition to allowing timely initiation of antiretroviral therapy of the HIV infection, early diagnosis also provides an opportunity to limit the spread of HIV from the infected individuals to the naive population.

Detection of anti-HIV antibodies as a marker of HIV exposure is the most widely used approach for serodiagnosis of this infection. Enzyme linked immunosorbent assay (ELISA) has been a preferred screening procedure in this regard [2]. However the labour intensive and time consuming format of the assay as well as the requirement of instrumentation and technical expertise has resulted in a shift from an ELISA based approach to rapid diagnostic tests (RDTs), particularly in resource constrained settings. While some studies have reported the performance of RDTs and ELISA to be comparable [3], results from others have raised concerns regarding sensitivity and specificity of the rapid assays [4–6].

With regard to HIV testing, two testing algorithms are commonly described: parallel and serial. While in parallel testing samples are tested simultaneously by two different tests, in serial testing they are tested by a first test, the results of which determine requirement of any further testing [7]. Thus for HIV testing strategies employing a serial testing algorithm, the selection of testing technologies as well as the order in which they are employed is crucial for obtaining accurate results. Since the first test is the screening test, it should be highly sensitive and the subsequent tests need to be highly specific so that all true negative test results are identified as negative and false positives do not occur. In India, the voluntary counselling and testing (VCT) facilities are employing strategy/algorithm III for diagnosis of HIV infection as per the guidelines laid by the National AIDS (acquired immunodeficiency syndrome) Control Organization (NACO) [8].

In the present study the authors have evaluated the performance of the RDT being used as the first/screening test in serial testing based algorithm for HIV diagnosis being followed at the VCT centre of a tertiary care health facility and compared it with the standard ELISA based approach for screening of HIV infection. In addition, all the positive results by the 3 RDTs and by ELISA were confirmed by a confirmatory test (western blot) to identify any false positives that may have occurred.

## 2. Materials and Methods

### 2.1. Study Population

This study is from the VCT facility of a tertiary care teaching hospital in North India. Sera from 787 consecutive patients tested at the VCT centre in September-October 2012 were included in the analysis.

Patients enrolled at the VCT facility first underwent a pretest counselling, following which a written informed consent was obtained for HIV testing and blood sample collected by trained technical personnel.

### 2.2. Evaluation Protocol

Sera were separated and tested by serial testing algorithm whereby samples reactive by the first test were subsequently tested by the second and third tests to confirm the positive result. The first test employed was SD Bioline HIV-1/2 3.0 (SD Biostandard Diagnostics Private Limited, Gurgaon, Haryana, India), a lateral flow immunochromatographic assay. Sera nonreactive by the first test were considered negative for anti-HIV antibodies whereas those that were reactive were subsequently tested with both Pareekshak HIV-1/2 Triline card test (Bhat Bio-Tech India Private Limited, Bangalore, Karnataka, India), lateral flow immunochromatographic assay, and Pareekshak HIV 1/2 rapid test kit (Trispot) (Bhat Bio-Tech India Private Limited, Bangalore, Karnataka, India), immunoconcentration based assay. All the specimens were processed as per instructions in the kit insert. In the second phase of this evaluation, all the sera (both HIV reactive and nonreactive) were retested by Microlisa-HIV (J. Mitra and Company Private Limited, New Delhi, India), an enzyme immunoassay based on the principle of indirect ELISA. The evaluation was conducted in a blinded fashion with the RDTs and ELISA performed by different technical personnel and the status of the sera as per the RDT based algorithm not revealed to the personnel performing the ELISA. All the samples reactive by Microlisa-HIV were retested by the same ELISA kit to confirm the result. All samples that were ELISA positive were confirmed by western blot (J. Mitra and Company Private Limited, New Delhi, India), as were the ones that were positive by RDTs alone. The serostatus as determined by western blot was considered as the final result. The characteristics of the RDTs and ELISA employed in this analysis are summarized in Table 1.

### 2.3. Statistical Analysis

The sensitivity and specificity calculations and estimation of negative and positive predictive values of the first/screening RDT were done by comparing its performance with Microlisa-HIV (reference technique). Sensitivity of a test is defined as the ability to correctly identify the infected individuals; specificity as the ability to correctly identify the uninfected individuals; negative predictive value as the proportion of those with a negative test result who are uninfected and positive predictive value as the proportion of those with a positive test result who are actually infected. Sensitivity was calculated as true positives/(true positives + false negatives) × 100; specificity as true negatives/(true negatives + false positives) × 100; negative predictive value as true negatives/(true negatives + false negatives) × 100 and positive predictive value as true positives/(true positives + false positives) × 100.

## 3. Results

All the 787 sera were tested for anti-HIV antibodies by at least one rapid test (SD Bioline HIV-1/2 3.0). Thirty-six serum samples were reactive by the first test. On subsequent evaluation of all the 787 samples by Microlisa-HIV, 40 HIV reactive samples were identified (all confirmed as positive by western blot), 9 of which had been reported as nonreactive by SD Bioline HIV-1/2 3.0. Thus the first RDT had missed 9 (22.5%) HIV reactive samples (also confirmed to be positive by western blot) and its sensitivity on comparison with ELISA was 77.5%. In addition, SD Bioline HIV-1/2 3.0 registered 5 false positive results (negative by ELISA and western blot) giving a specificity of 99.3%. The negative and positive predictive values of SD Bioline HIV-1/2 3.0 were 98.8% and 86.1%, respectively. The diagnostic performance of SD Bioline HIV-1/2 3.0 and its performance characteristics in comparison to Microlisa-HIV are summarized in Tables 2 and 3, respectively.

Since Pareekshak HIV-1/2 Triline card test and Pareekshak HIV 1/2 rapid test kit (Trispot) were used to evaluate only those samples that were reactive by the first test, their overall sensitivity and specificity could not be assessed though both the tests picked up all 31(100%) of ELISA reactive serum samples tested by them. Like SD Bioline HIV-1/2 3.0, both the tests also registered false positive results (5 false positives by Pareekshak HIV-1/2 Triline card test and 4 false positives by Pareekshak HIV 1/2 rapid test kit (Trispot)). An overview of the reactive results obtained by the 3 RDTs and their subsequent status as per Microlisa-HIV is provided in Table 4.

## 4. Discussion

Both ELISA and RDTs are widely employed immunological assays for serodiagnosis of HIV infection [9]. Discrepancy between results obtained by the two techniques is common [10]. Some studies suggest that the diagnostic performance of RDTs is comparable to that of ELISA [3]. However, in our evaluation RDT based algorithm employing SD Bioline HIV-1/2 3.0 as the initial test fared poorly compared to ELISA and missed a large proportion of HIV infections. Inferior performance of RDTs in comparison to ELISA has also been reported in another study from India where both the modalities were used to screen healthy blood donors for HIV infection and the RDT used missed 17 of 30 samples confirmed reactive by ELISA [2]. This discordance may possibly be due to low antibody titres especially in recent infections where the levels may well be below the detection limit of RDTs but are picked up by the more sensitive enzyme immunoassay and its spectrophotometric format of result analysis. Based on our findings we suggest that, with serial testing based algorithms wherein a negative result from a single initial RDT is considered sufficient to classify a sample as HIV nonreactive, false negative results may occur quite often leading to unknown silent transmission of HIV in the population.

Since the primary purpose of this study was not to compare the performance of different RDTs with each other and we have strictly adhered to the protocol of a serial testing algorithm, we cannot make recommendations regarding the use of any one RDT over others as the initial test. We also do not deny that the same RDT may perform differently under different field conditions, since on literature review we found studies that show the same rapid test brand as employed in our analysis to be 100% sensitive and equivalent in its diagnostic performance characteristics to Microlisa-HIV [11]. Thus while we cannot rule out the possibility that an RDT of the same or of a different test brand might perform better, as discussed above, the theoretical probability of an RDT approaching the sensitivity of an ELISA is meagre especially for low titre samples as seen in the diagnostic window period.

As with other studies, we also observed false positive results with RDTs [12]. False positive results with RDTs are a matter of concern since the relative ease of performance of these assays outside the laboratories increases the likelihood of HIV testing without proper counselling, which further leads to poor understanding on the part of the patients receiving a reactive report that they may or may not be positive [13]. In our study, the false positives with RDTs are definitely not due to cross-reactivity since all these samples were nonreactive by ELISA. While the cause of these false positives is not exactly known, the common reasons could be technical errors, mislabelling of samples, problems with components of the test devices, and subjective and ambiguous interpretation of faint bands as positive when the test sample is actually negative [14, 15]. A recent study has also pointed out the role of variation in specificity of HIV RDTs over time and geographic location as a possible cause of higher than previously encountered false positive HIV results [16].

A major constraint on the application of ELISA in VCT programs despite its high sensitivity has been its longer turnaround time and with studies revealing that a number of patients undergoing HIV testing do not return to the VCT centres to collect their reports [17, 18], RDTs seem to offer an excellent option with test results being available to the patients on the same visit. Though use of RDTs can increase the proportion of patients gaining access to HIV antibody test results [19], their use as the only screening test in VCT programs cannot be justified keeping in view the possibility of missing early infections as well as concerns regarding reporting false positive results.

A practical approach in the present scenario could be a serial RDT based testing algorithm to despatch a preliminary report to the patient followed by testing of all samples by ELISA to identify any false negative and false positive results. In resource limited settings like India, where serial testing algorithms are cost effective and putting up both RDTs and ELISA for all samples might not be economically feasible, efforts should be directed towards utilizing RDTs that simultaneously detect p24 antigen, so that early HIV infections are not missed, and confirming at least all the samples positive for anti-HIV antibodies by RDTs with a subsequent conventional ELISA and western blot to reduce the frequency of persons receiving false positive results.

## 5. Conclusion

Our study highlights that ELISA is a good screening assay for HIV infection. The performance of RDTs in comparison to ELISA is suboptimal and RDT based serial testing algorithm cannot parallel the testing accuracy of an ELISA based approach. While false negatives by RDTs increase the proportion of HIV reactive individuals receiving negative reports, false positives by RDTs are a matter of ethical concern. The diagnostic limitations of RDTs can be overcome by possible inclusion of ELISA as a second screening assay, employing RDTs additionally detecting p24 antigen as screening assays, and confirmation of reactive samples by western blot to reduce false negative and false positive results, respectively.

## Figures and Tables

**Table 1:** Characteristics of human immunodeficiency virus (HIV) tests as per kit literatures.

Test	Principle	Specimen volume required	Antigen employed	Time to test result	Specimen type	Sensitivity	Specificity
SD Bioline HIV-1/2 3.0	Lateral flow immunochromatography	10 *μ*L	gp 41 and p24 for HIV-1 and gp 36 for HIV-2	5–20 minutes	Serum, plasma	Not specified	Not specified
Pareekshak HIV-1/2 Triline card test	Lateral flow immunochromatography	10 *μ*L	gp 41 and C terminus of gp 120 for HIV-1 and gp 36 for HIV-2	20 minutes	Serum, plasma	100%	99.49%
Pareekshak HIV 1/2 rapid test kit (Trispot)	Immunoconcentration	2 drops	HIV 1 and HIV-2 r (recombinant)-proteins	Immediate	Serum, plasma	100%	100%
Microlisa-HIV	Indirect ELISA	10–20 *μ*L	gp 41 and C terminus of gp 120 for HIV-1 and gp 36 for HIV-2	>1.5 hours	Serum, plasma	100%	99.5%

HIV: human immunodeficiency virus; ELISA: enzyme linked immunosorbent assay.

**Table 2:** Diagnostic performance of SD Bioline HIV-1/2 3.0 in comparison to Microlisa-HIV (reference standard).

Test 1 (SD Bioline HIV-1/2 3.0)	Microlisa-HIV	
	Reactive	Nonreactive
Reactive	31	5
Nonreactive	9	742

HIV: human immunodeficiency virus.

**Table 3:** Sensitivity, specificity, and predictive values of SD Bioline HIV-1/2 3.0 (taking Microlisa-HIV as the reference).

Test	Sensitivity	Specificity	Negative predictive value	Positive predictive value
SD Bioline HIV-1/2 3.0	77.5%	99.3%	98.8%	86.1%

HIV: human immunodeficiency virus; ELISA: enzyme linked immunosorbent assay.

**Table 4:** Comparison of reactive results of rapid test kits with Microlisa-HIV (reference standard).

Test employed	Total number of tests performed	Number of samples positive	Number of samples reactive by Microlisa-HIV	Number of samples nonreactive by Microlisa-HIV
SD Bioline HIV-1/2 3.0	787	36	31	5
Pareekshak HIV-1/2 Triline card test	36	36	31	5
Pareekshak HIV 1/2 rapid test kit (Trispot)	36	35	31	4

HIV: human immunodeficiency virus.
